# Integrative Multi-Omics Analysis Reveals HNRNPLL as a Potential Biomarker Associated with Hepatocellular Carcinoma Progression

**DOI:** 10.3390/metabo16040234

**Published:** 2026-03-31

**Authors:** Xiaojing Wang, Bin Li, Kun Li, Dan Wan, Nanbin Liu

**Affiliations:** 1 Department of Pathology, The Second Affiliated Hospital of Xi’an Jiaotong University, Xi’an 710004, China; wangxiaojing1990@xjtu.edu.cn (X.W.); wdd1314@xjtu.edu.cn (D.W.); lnb10731@xjtu.edu.cn (N.L.); 2 Department of Stomatology, Hunan University of Medicine, Huaihua 410208, China

**Keywords:** liver hepatocellular carcinoma, HNRNPLL, prognosis, chemotherapy, immunotherapy

## Abstract

**Background**: Heterogeneous nuclear ribonucleoprotein L-like (HNRNPLL) is an RNA-binding protein involved in alternative splicing and immune regulation; however, its role in liver hepatocellular carcinoma (LIHC) remains unclear. **Methods**: We performed integrative multi-omics analyses using data from TCGA, GEO, and the Human Protein Atlas to evaluate the expression patterns, prognostic value, and potential biological functions of HNRNPLL. Functional enrichment and immune-related analyses were conducted to explore associated pathways. Experimental validation was performed in LIHC cell lines using Western blotting, RT-qPCR, CCK-8, colony formation, and Transwell assays, along with a xenograft mouse model. **Results**: HNRNPLL was significantly upregulated in LIHC at both transcriptomic and proteomic levels and was associated with advanced clinicopathological features and poor overall survival. Multivariate Cox regression analysis identified HNRNPLL as an independent prognostic factor. Enrichment analyses suggested that HNRNPLL-related genes are mainly involved in cell cycle regulation, mitotic progression, epithelial–mesenchymal transition, and immune-related pathways. In addition, HNRNPLL expression was correlated with immune cell infiltration, tumor mutational burden, microsatellite instability, ferroptosis-related genes, and m6A methylation regulators. Functional experiments demonstrated that HNRNPLL knockdown suppressed proliferation, migration, and invasion of liver cancer cells and inhibited tumor growth in vivo. **Conclusions**: These findings suggest that HNRNPLL may act as a potential regulator of LIHC progression and is associated with tumor-related biological processes and immune features. HNRNPLL may serve as a candidate biomarker for prognosis and a potential therapeutic target in LIHC, although further mechanistic studies are required.

## 1. Introduction

Hepatocellular carcinoma (HCC, also referred to as liver hepatocellular carcinoma, LIHC) is one of the most common and lethal malignancies worldwide [1]. Despite advances in surgical resection, local ablation, and systemic therapies, the overall prognosis of patients with advanced LIHC remains poor due to late diagnosis, high recurrence rates, and limited therapeutic options [2,3,4]. Therefore, identifying novel biomarkers and therapeutic targets is essential for improving early diagnosis and treatment outcomes.

RNA-binding proteins (RBPs) play critical roles in post-transcriptional regulation, including mRNA splicing, stability, transport, and translation [5,6]. Increasing evidence indicates that dysregulation of RBPs contributes to tumor initiation and progression [7,8]. Among these, the heterogeneous nuclear ribonucleoprotein (hnRNP) family has been widely implicated in cancer-related processes such as cell proliferation, apoptosis, epithelial–mesenchymal transition (EMT), and immune regulation [9,10].

Heterogeneous nuclear ribonucleoprotein L-like (HNRNPLL) is a member of the hnRNP family that is primarily involved in alternative splicing and RNA metabolism [11]. Previous studies have demonstrated that HNRNPLL plays an important role in lymphocyte differentiation and immune homeostasis [12]. In addition, emerging evidence suggests that HNRNPLL may participate in tumor-related processes, such as regulating alternative splicing events associated with cancer progression [13,14,15,16]. However, the role of HNRNPLL in LIHC remains largely unclear.

With the rapid development of high-throughput sequencing technologies, integrative multi-omics analyses have become powerful approaches for systematically investigating cancer-related genes [17]. Public databases such as The Cancer Genome Atlas (TCGA), Gene Expression Omnibus (GEO), and the Human Protein Atlas (HPA) provide valuable resources for exploring gene expression patterns, prognostic significance, and potential biological functions [18]. However, bioinformatics findings alone are often insufficient and require experimental validation to confirm their biological relevance [19].

In this study, we performed a comprehensive analysis of HNRNPLL using publicly available datasets, combined with experimental validation in LIHC models. We systematically evaluated the expression patterns and prognostic value of HNRNPLL in LIHC and explored its potential biological functions through enrichment and immune-related analyses. Furthermore, in vitro and in vivo experiments were conducted to investigate the functional role of HNRNPLL in LIHC. This study aims to provide new insights into the potential role of HNRNPLL in LIHC and explore its value as a candidate biomarker and therapeutic target.

## 2. Materials and Methods

### 2.1. Data Acquisition and Processing

RNA sequencing data and corresponding clinical information for pan-cancer were obtained from The Cancer Genome Atlas (TCGA) database (https://portal.gdc.cancer.gov/, accessed on 1 January 2026) [20]. Gene expression data were normalized to transcripts per million (TPM) values and log2-transformed for subsequent analyses. Samples lacking complete clinical information were excluded.

Spatial transcriptomic data from four pan-cancer samples (IDs: BRCA_GSE176078-1160920F, CRC_NC-CRC_P6, HCC_Pmid34919432-HCC-2L, HCC_Pmid34919432-HCC-4L) were retrieved from the SpatialTME database (https://www.spatialtme.yelab.site/#, accessed on 1 January 2026) [21]. Gene expression datasets GSE37182, GSE45267, GSE76427, and GSE112790 were downloaded from the Gene Expression Omnibus (GEO) database (https://www.ncbi.nlm.nih.gov/geo/, accessed on 1 January 2026) and used for external validation.

Protein expression and immunohistochemistry data were obtained from the Human Protein Atlas (HPA) database (https://www.proteinatlas.org/, accessed on 1 January 2026) [22].

Data visualization and statistical analyses were performed using R software (version 4.2.0) with packages including ‘ggplot2’, ‘limma’, ‘survival’, and ‘pROC’.

### 2.2. Patient and Tissue Samples

Five pairs of LIHC tissues and adjacent non-tumor tissues were collected from patients at the Second Affiliated Hospital of Xi’an Jiaotong University. All patients provided written informed consent. This study was approved by the institutional ethics committee and conducted in accordance with the Declaration of Helsinki.

### 2.3. Cell Line Culture

The human liver cancer cell lines HepG-2 and Huh7 were purchased from the Cell Bank of the Chinese Academy of Sciences. Cells were routinely cultured in Dulbecco’s Modified Eagle Medium (DMEM, Gibco, CA, USA, Cat#11965-092) containing 10% heat-inactivated fetal bovine serum (FBS, Corning, NY, USA, Cat#35-010-CV) and 1% penicillin–streptomycin antibiotic solution (Beyotime, Shanghai, China, Cat#C0222). All cells were maintained at 37 °C in a humidified incubator with a 5% CO_2_ atmosphere.

### 2.4. Genomic Alteration Analysis

Genomic alterations of HNRNPLL were analyzed using the cBioPortal database (https://www.cbioportal.org/, accessed on 1 January 2026) [23]. Mutation frequency, mutation types, and mutation sites were evaluated across different cancer types.

Tumor mutational burden (TMB) and microsatellite instability (MSI) data were obtained from TCGA [24]. Correlation analyses between HNRNPLL expression and TMB/MSI were performed using Spearman correlation.

Copy number variation (CNV) data were analyzed using the GSCA database (https://guolab.wchscu.cn/GSCA/, accessed on 1 January 2026) [25]. Somatic mutation data were visualized using the ‘maftools’ R package (version 4.0.3) [26].

### 2.5. Prognostic and Diagnostic Analysis

The diagnostic value of HNRNPLL was evaluated using receiver operating characteristic (ROC) curves, and the area under the curve (AUC) was calculated using the ‘pROC’ package (version 4.0.3).

Survival analyses, including overall survival (OS), disease-specific survival (DSS), and progression-free interval (PFI), were conducted using Kaplan–Meier analysis and Cox proportional hazards regression models [27].

Patients were divided into high- and low-expression groups based on the median expression level of HNRNPLL. Hazard ratios (HRs) and 95% confidence intervals (CIs) were calculated.

### 2.6. Clinicopathological Correlation of HNRNPLL in GBM and LIHC

To gain deeper insights into the association between HNRNPLL expression and clinical–pathological features in GBM and LIHC, logistic regression analyses were performed. Wilcoxon rank-sum or Kruskal–Wallis tests were employed to elucidate potential correlations between HNRNPLL expression levels and diverse clinical–pathological parameters, including pathologic stage, histologic grade, tumor status, age, gender, race, BMI, residual tumor, histologic grade, AFP, prothrombin time (PT), Child–Pugh grade, Ishak fibrosis score, vascular invasion, adjacent hepatic tissue inflammation, IDH status, and Kamofsky performance score. Statistically significant variables were visualized using the ‘ggplot2’ package.

### 2.7. Differential Expression and Enrichment Analysis

Differentially expressed genes (DEGs) between high- and low-HNRNPLL expression groups were identified using the ‘limma’ package (version 3.65.5). Genes with |log1.5 fold change| > 1 and adjusted *p* value (*p*.adj) < 0.05 were considered statistically significant.

Gene Ontology (GO) and Kyoto Encyclopedia of Genes and Genomes (KEGG) enrichment analyses were performed using the ‘clusterProfiler’ package (version 4.18.4).

Gene Set Enrichment Analysis (GSEA) was conducted using predefined gene sets, and pathways with a false discovery rate (FDR) < 0.001 and *p*.adj < 0.001 were considered significantly enriched [28].

### 2.8. Identification of HNRNPLL-Related Genes and PPI Networks in GBM and LIHC

We individually evaluated the top 30 genes most strongly correlated with HNRNPLL in GBM and LIHC, respectively, and visualized them using the ‘ggplot2’ package (version 4.0.3). Additionally, we explored the PPI network of HNRNPLL using the STRING database (https://cn.string-db.org/, accessed on 1 January 2026) [29]. For both GBM and LIHC, we selected the top 10 genes with the highest connectivity within the co-expression network, as well as the top 10 genes with the most significant interaction nodes in the PPI network. To investigate the pan-cancer associations of HNRNPLL and its related genes, we employed the Tumor Immune Assessment Resource 2.0 (TIMER2) (http://timer.cistrome.org/, accessed on 1 January 2026) [30]. We further analyzed the interrelationships among the aforementioned genes in GBM and LIHC and visualized them using the ‘corrplot’ package (version 0.95).

### 2.9. Prognostic Significance of HNRNPLL Co-Expressed Genes in LIHC

We investigated the prognostic role of HNRNPLL co-expressed genes in LIHC, examined their expression across different tumor stages, and utilized GSCALite (https://guolab.wchscu.cn/GSCA, accessed on 1 January 2026) to identify signaling pathways co-enriched by HNRNPLL and its associated prognostic genes in GBM and LIHC [31].

### 2.10. Immune Infiltration Analysis

The relative proportions of tumor-infiltrating immune cells were estimated using the CIBERSORT algorithm. The association between HNRNPLL expression and immune cell infiltration was evaluated using Spearman correlation analysis of data from the TISIDB online database (http://cis.hku.hk/TISIDB/index.php, accessed on 1 January 2026) [32].

Immune scores, stromal scores, and ESTIMATE scores were calculated using the ‘ESTIMATE’ R package (version 1.1.1) [33].

### 2.11. Single-Cell Expression Analysis of HNRNPLL in LIHC

We obtained single-cell data in .h5 format and corresponding LIHC annotation information from the Tumor Immune Single-Cell Hub (TISCH) database (http://tisch.compbio.cn/, accessed on 1 January 2026) [34]. The single-cell data were processed and analyzed using the ‘MAESTRO’ and ‘Seurat’ packages (version 4.0.3), with cell clustering and subset identification achieved via the t-distributed stochastic neighbor embedding (t-SNE) method.

### 2.12. Immunotherapy and Chemotherapy Responses Associated with HNRNPLL in LIHC

We obtained RNA sequencing profiles and corresponding clinical data for LIHC from the TCGA dataset. Subsequently, we applied the Tumor Immune Dysfunction and Exclusion (TIDE) algorithm to predict potential responses to immune checkpoint inhibitor (ICI) therapy. In addition, we utilized the Genomic Drug Sensitivity in Cancer (GDSC) and Cancer Therapy Response Portal (CTRP) databases to predict chemotherapy responses in LIHC patients with high and low HNRNPLL expression levels [35]. Drug sensitivity prediction was performed using the ‘prophetic’ package (version 4.0.3), with the half-maximal inhibitory concentration (IC50) estimated via ridge regression analysis [36].

### 2.13. Association of HNRNPLL with Ferroptosis- and m6A Methylation-Related Genes in GBM and LIHC and Molecular Docking Analysis

We performed gene localization analysis of HNRNPLL using the Coremine Medical Ontology Information Retrieval Platform (www.coremine.com/medical/, accessed on 1 January 2026) to identify its top 10 potential regulatory molecules [37]. Structural files for HNRNPLL were downloaded from the PDB database (https://www.rcsb.org/, accessed on 1 January 2026) to obtain the target protein structure file [38]. Structural files for active compounds were obtained from the PubChem database (https://pubchem.ncbi.nlm.nih.gov/, accessed on 1 January 2026), and the CB-Dock2 platform was used to optimize structures (by removing water molecules and ligands) and perform molecular docking simulations. This approach assessed the binding interactions between the receptor protein and small-molecule ligands while balancing charges on the receptor protein [39].

Ferroptosis is an unconventional form of regulated cell death characterized by iron-dependent accumulation of lethal peroxides on membranes, involving iron overload, free radical propagation, antioxidant system impairment, and lipid peroxidation [40]. N6-methyladenosine (m6A) methylation is one of the most common RNA modifications in mRNA and non-coding RNAs, and dysregulation of enzymes involved in m6A methylation may contribute to various diseases [41].

We investigated the correlations between HNRNPLL and ferroptosis, as well as m6A methylation-associated genes in GBM and LIHC. Ferroptosis-related genes were collected from a systematic analysis of ferroptosis mechanisms and their significance in malignancies by Ze-Xian Liu et al. [42]. m6A methylation-associated genes were obtained from Yongsheng Li et al.’s analysis of the molecular features and clinical implications of m6A methylation modulators across 33 cancer types [43].

### 2.14. Western Blotting

Proteins were extracted from tissues or cells using RIPA lysis buffer (Beyotime) on ice for 20 min. After centrifugation at 13,000× *g* for 20 min, the protein concentration in the supernatant was measured via a BCA assay. Equal protein amounts were loaded for separation on 10% SDS-PAGE gels and transferred to PVDF membranes. Following blocking with 5% non-fat milk, membranes were probed overnight at 4 °C with primary antibodies against HNRNPLL (Proteintech, *Cat#*26769-1-AP, 1:5000) and GAPDH (Cell Signaling Technology, *Cat#*2118, 1:2000) and then with appropriate HRP-conjugated secondary antibodies for 2 h at room temperature. Signals were detected by ECL and band intensities were quantified with ImageJ (version 8.0). HNRNPLL expression levels were normalized to GAPDH. Data shown are representative of three independent experiments.

### 2.15. Quantitative Real-Time PCR (RT-qPCR)

Total RNA was extracted from tissue samples and transfected cells using TRIzol™ reagent (Invitrogen, *Cat#*15596026CN) according to the manufacturer’s protocol. cDNA was then synthesized from the isolated RNA utilizing a PrimeScript RT Reagent Kit (Takara, Tokyo, Japan). Quantitative real-time PCR (RT-qPCR) was performed with the Takara SYBR Premix Ex Taq II Kit on a real-time PCR system. The expression levels of target mRNAs were calculated via the 2^−ΔΔCt^ method, normalized against the endogenous control GAPDH. All experiments were performed with three independent biological replicates (*n* = 3). The nucleotide sequences of the primers used are listed below:

HNRNPLL-forward: CTACAAGCAAAAGGATCACTCG

HNRNPLL-reverse: CCACTGTAATTGGATAAAGCGG

GAPDH-forward: TGTGGGCATCAATGGATTTGG

GAPDH-reverse: ACACCATGTATTCCGGGTCAAT

### 2.16. Silencing of HNRNPLL by Small Interfering RNA (siRNA)

To achieve HNPNPLL knockdown, we utilized three specific siRNAs and a negative control (NC) siRNA (Qingke). In preparation, HepG-2 and Huh7 cells were seeded onto 6-well plates (2 × 10^5^ cells/well) and cultured overnight. The following day, cells were transfected with 100 nM of either the selected HNRNPLL-targeting siRNA (si-HNRNPLL-3) or the NC siRNA, employing Lipofectamine 2000 (Invitrogen, Cat#2477064) as the delivery reagent. A 6 h transfection period was used, after which the medium was changed to standard growth medium. To confirm successful knockdown, cells were collected 24 h post-transfection for evaluation of HNRNPLL mRNA and protein levels via RT-qPCR and Western blot.

### 2.17. Colony-Formation

Post-transfection, cells were plated at 1000 cells/well in 6-well plates and cultured for 7 days under standard conditions (37 °C, 5% CO_2_) to form colonies. Colonies were then fixed with 4% paraformaldehyde (Biosharp, Cat#BL539A) for 30 min, stained with crystal violet (Beyotime, Cat#C0121-500mL) for 20 min, and air-dried prior to imaging and quantification. The experiment was performed in triplicate wells and repeated three times independently.

### 2.18. Cell Proliferation

To assess proliferation, cells transfected with si-HNRNPLL were seeded onto 96-well plates at a density of 1000 cells per well. Cell viability was measured at 0, 24, 48, and 72 h post-seeding using the Cell Counting Kit-8 (CCK-8, Beyotime, Cat#C0038) according to the manufacturer’s instructions. Briefly, at each time point, 10 µL of CCK-8 reagent was added to each well, followed by incubation at 37 °C for 1–4 h. The absorbance (optical density, OD) at 450 nm was then measured using a multifunctional microplate reader (Thermo Fisher Scientific, Waltham, MA, USA). Each condition was assayed in technical triplicate, and the entire experiment was independently repeated three times (n = 3).

### 2.19. Transwell Cell Migration and Invasion

Cell migratory and invasive capacities were assessed using Transwell chambers (8 µm pore size; Corning, Corning, NY, USA). For the migration assay, successfully transfected cells (5 × 10^5^) in 200 µL of serum-free medium were seeded into the upper chamber, while, for the invasion assay, the chamber was pre-coated with 60 µL of Matrigel (Beyotime, Cat#C0371-10mL, diluted 1:8 in serum-free medium) and polymerized at 37 °C for 1–3 h prior to cell seeding. In both assays, the lower chamber contained 700 µL of DMEM with 10% FBS as a chemoattractant. After 24 h of incubation, non-migratory/non-invasive cells on the upper membrane surface were removed with a cotton swab. Cells that had traversed the membrane were fixed with 4% paraformaldehyde for 20 min and stained with 0.1% crystal violet for 20 min. Membranes were photographed under a microscope, and cells from multiple random fields were counted. Each experiment was conducted in triplicate and independently repeated three times (n = 3).

### 2.20. Nude Mouse Tumor Model

To establish xenograft models, male nude mice (n = 5 per group) received subcutaneous injections of 5 × 10^5^ HepG2-sh-NC or HepG2-sh-HNRNPLL cells. An in vitro inoculation experiment was performed under isoflurane inhalation anesthesia 2%. Tumor growth was monitored every other day via bi-dimensional caliper measurements; tumor volume was estimated using the formula V = 0.5 × length × width^2^. At the experimental endpoint (day 15), mice were humanely euthanized by gradual exposure to carbon dioxide (CO_2_) at a flow rate of 20% of the chamber volume per minute, and the tumors were surgically removed, followed by immediate measurement of dimensions and weight, as well as photographic recording. All animal experiments were conducted in accordance with the guidelines approved by the Bioethics Committee of the Medical Department of Xi’an Jiaotong University. We adhere to the animal research: reporting of in vivo experiments (ARRIVE) guidelines and the guidelines for the welfare and use of animals in cancer research.

### 2.21. Immunohistochemistry

For Ki67 immunohistochemistry, paraffin-embedded tissue sections (4 μm) were deparaffinized and rehydrated. Antigen retrieval was performed by heating in citrate buffer (pH 6.0) for 15 min. Endogenous peroxidase activity was blocked with 3% H_2_O_2_. Sections were then incubated overnight at 4 °C with a primary antibody against Ki67 (Rabbit monoclonal, Abcam, Cambridge, UK, Cat# ab15580) at a dilution of 1:200. Subsequently, sections were incubated with HRP-conjugated secondary antibody (Anti-Rabbit IgG, Proteintech, Cat# PK1009) for 1 h at room temperature. The reaction was visualized using DAB (3,3′-diaminobenzidine) and counterstained with hematoxylin.

### 2.22. Statistics

All data were analyzed using R software (version 4.0.3) and GraphPad Prism (version 8.0). Normality was assessed using the Shapiro–Wilk test and homogeneity of variances was examined using Levene’s test. Data following a normal distribution are presented as the mean ± standard deviation (SD), whereas non-normally distributed data are presented as the median and interquartile range (IQR). For comparisons between two groups, independent Student’s *t*-tests were used for normally distributed data, and Mann–Whitney *U* tests were applied otherwise. For comparisons involving more than two groups, one-way analyses of variance (ANOVAs) were conducted. When significant main effects or interactions were detected in the ANOVA, post-hoc pairwise comparisons were performed using Tukey’s honestly significant difference (HSD) test to control for type I error inflation due to multiple comparisons. All experiments were repeated in at least three independent biological replicates. For in vivo studies, each group consisted of a minimum of five animals (n ≥ 5). The threshold for statistical significance was set at *p* < 0.05 for all analyses. Post-hoc results are reported with adjusted *p*-values.

## 3. Results

### 3.1. Distribution of HNRNPLL in Healthy Human Organs and Tissues

**Figure A1:**
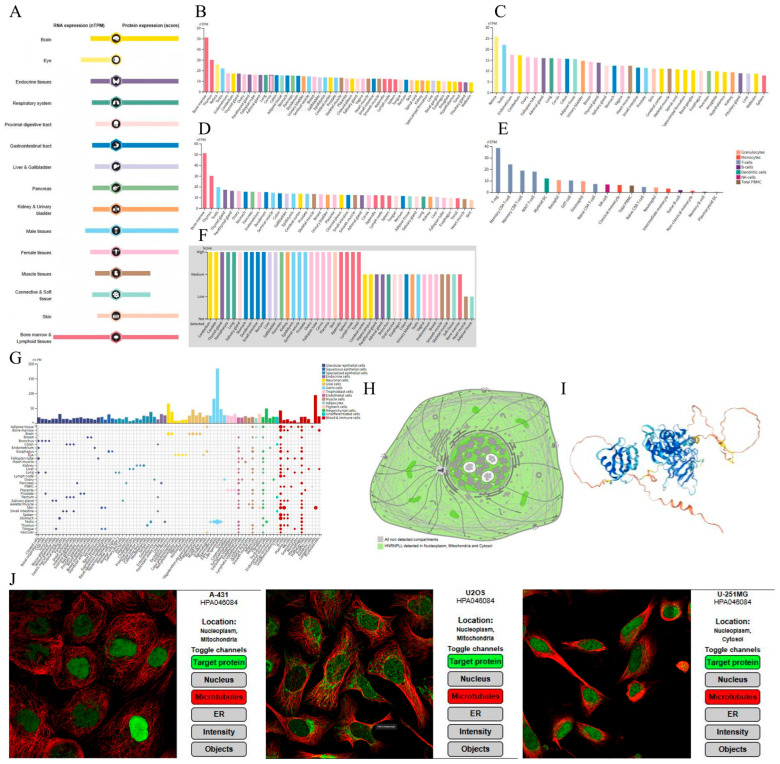
Expression of HNRNPLL in normal human organs and tissues. (**A**) Overview of HNRNPLL mRNA and protein expression in human organs and tissues. (**B**–**D**) Expression patterns of HNRNPLL mRNA in different tissues from consensus, GTEx, and HPA databases. (**E**) Expression patterns of HNRNPLL mRNA in various immune cell types from the HPA database. (**F**) Distribution of HNRNPLL protein expression scores across human organs in the HPA database. (**G**) Overview of HNRNPLL mRNA expression in diverse single-cell tissues. (**H**) Subcellular localization of HNRNPLL. (**I**) HNRNPLL protein structure. (**J**) Immunofluorescence visualization of HNRNPLL subcellular localization in the HPA database.

HNRNPLL mRNA is widely distributed across various human organs and tissues (Figure A1A). Analysis of the Consensus dataset reveals that HNRNPLL is predominantly expressed in the bone marrow, thymus, retina, testis, endometrium, cerebellum, thyroid gland, ovary, parathyroid gland, fallopian tube, and adrenal gland (Figure A1B). Investigation of the GTEx dataset indicates that HNRNPLL is primarily localized in the retina, testis, endometrium, cerebellum, ovary, fallopian tube, adrenal gland, lung, cervix, and colon (Figure A1C). Similarly, findings from the HPA dataset suggest that HNRNPLL is mainly distributed in the bone marrow, thymus, testis, thyroid gland, parathyroid gland, ovary, stomach, pancreas, endometrium, and duodenum (Figure A1D). Immune cell profiling revealed that HNRNPLL is notably enriched in Treg cells, memory CD4+ T cells, memory CD8+ T cells, MALT T cells, and myeloid dendritic cells, with relatively low immune cell specificity (Figure A1E). At the protein level, HNRNPLL protein showed moderate expression in the cerebellum, caudate, thyroid gland, nasopharynx, lung, salivary gland, stomach, duodenum, small intestine, rectum, liver, gallbladder, pancreas, kidney, epididymis, seminal vesicle, prostate, ovary, fallopian tube, cervix, placenta, skin, appendix, spleen, lymph node, and tonsil (Figure A1F). As illustrated in Figure A1G, the distribution of HNRNPLL gene expression exhibits considerable heterogeneity across different tissues and cell types, with notably elevated levels in germ cells of the testis—particularly in spermatocytes and early/late spermatids—as well as high expression in Langerhans cells and excitatory neurons of various organs. We also obtained information on HNRNPLL subcellular localization and protein structure features from the HPA database (Figure A1H–J). Immunofluorescence localization of HNRNPLL in A-431, U-2 OS, and U-251 MG cells was obtained, with green indicating the localization and intensity of HNRNPLL expression and red representing microtubule structures. This reveals that HNRNPLL is predominantly localized to the nucleoplasm, mitochondria, and cytosol.

### 3.2. HNRNPLL Is Significantly Elevated in LIHC and Various Other Malignancies

**Figure 1:**
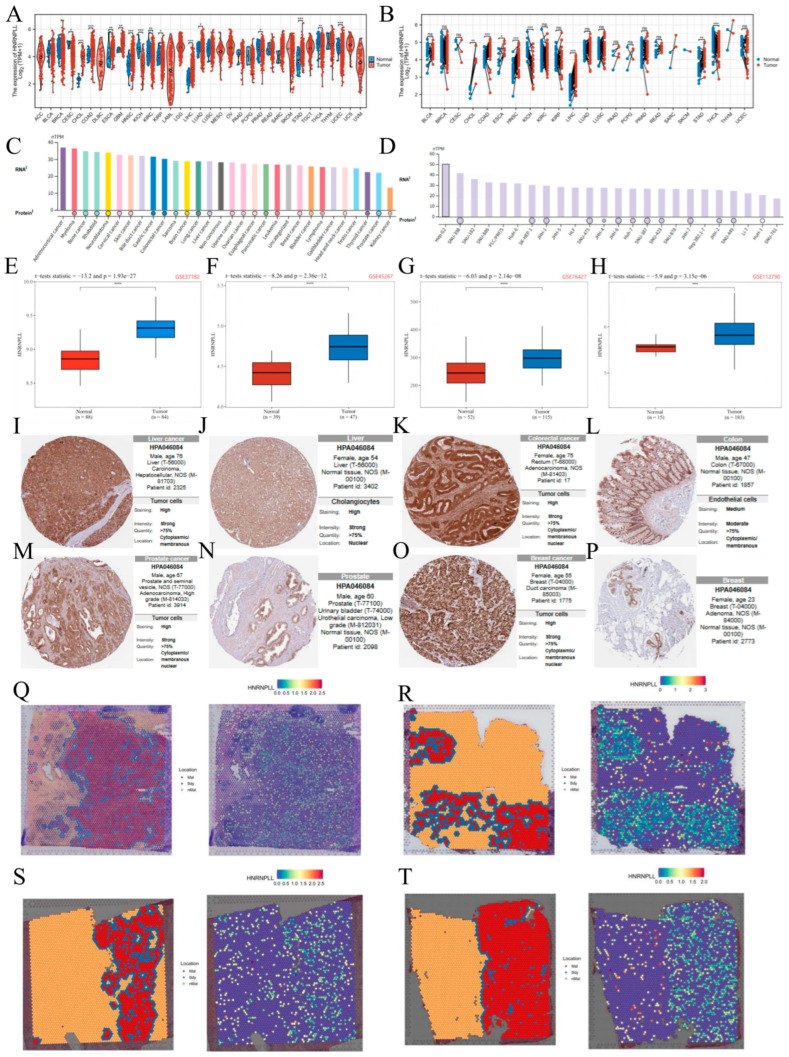
HNRNPLL expression landscape. (**A**,**B**) Display HNRNPLL expression levels in unmatched and matched pan-cancer tissues from the TCGA database. (**C**) mRNA expression of HNRNPLL across different cancer types. (**D**) mRNA expression of HNRNPLL in various pan-cancer cell lines. (**E**–**H**) HNRNPLL mRNA expression in LIHC based on GEO databases, using datasets GSE37182, GSE45267, GSE76427, and GSE112790. (**I**–**P**) HPA database IHC staining showing HNRNPLL protein expression in hepatocellular carcinoma, normal liver tissue, colorectal carcinoma, normal colon tissue, prostate carcinoma, normal prostate tissue, breast carcinoma, and normal breast tissue. (**Q**–**T**) Spatial distribution of normal tissue and tumor tissue in HCC_Pmid34919432-HCC-2L and HCC_Pmid34919432-HCC-4L tissue sections for BRCA_GSE176078-1160920F, CRC_NC-CRC_P6, HCC_Pmid34919432-HCC-2L, and HCC_Pmid34919432-HCC-4L tissue sections, along with the corresponding spatial distribution of HNRNPLL. (ns: no significance; * *p* < 0.05; ** *p* < 0.01; *** *p* < 0.001, **** *p* < 0.0001).

After analyzing the pan-cancer data from TCGA, we examined HNRNPLL expression levels across 15 unpaired and 8 paired cancer types. HNRNPLL expression was elevated in cholangiocarcinoma (CHOL), colorectal adenocarcinoma (COAD), esophageal carcinoma (ESCA), head and neck squamous cell carcinoma (HNSC), LIHC, and stomach adenocarcinoma (STAD), whereas it was decreased in kidney chromophobe (KICH) and thyroid carcinoma (THCA) (Figure 1A,B). In addition, analysis of the HPA dataset revealed that HNRNPLL mRNA was predominantly detected in adrenocortical cancer, myeloma, and bone cancer cell lines, while the protein was observed in myeloma, bone cancer, and rhabdoid tumors, among others (Figure 1C). Examination of cancer cell lines showed widespread distribution of HNRNPLL in lines including Hep-G2, SNU-398, and SNU-182 (Figure 1D). Consistent with these findings, our evaluation of four GEO datasets demonstrated HNRNPLL upregulation in LIHC and COAD (Figure 1E–H). To further validate the protein expression characteristics of HNRNPLL, we examined IHC staining images from the HPA database, revealing increased HNRNPLL levels in LIHC, colorectal cancer (CRC), prostate cancer, and breast cancer (BRCA) compared with normal tissues (Figure 1I–P). Analysis of spatial transcriptomics data showed that HNRNPLL expression levels in BRCA, CRC, and LIHC were predominantly localized within tumor regions in section-based studies (Figure 1Q–T).

### 3.3. HNRNPLL Gene Mutation Landscape in Pan-Cancer

**Figure A2:**
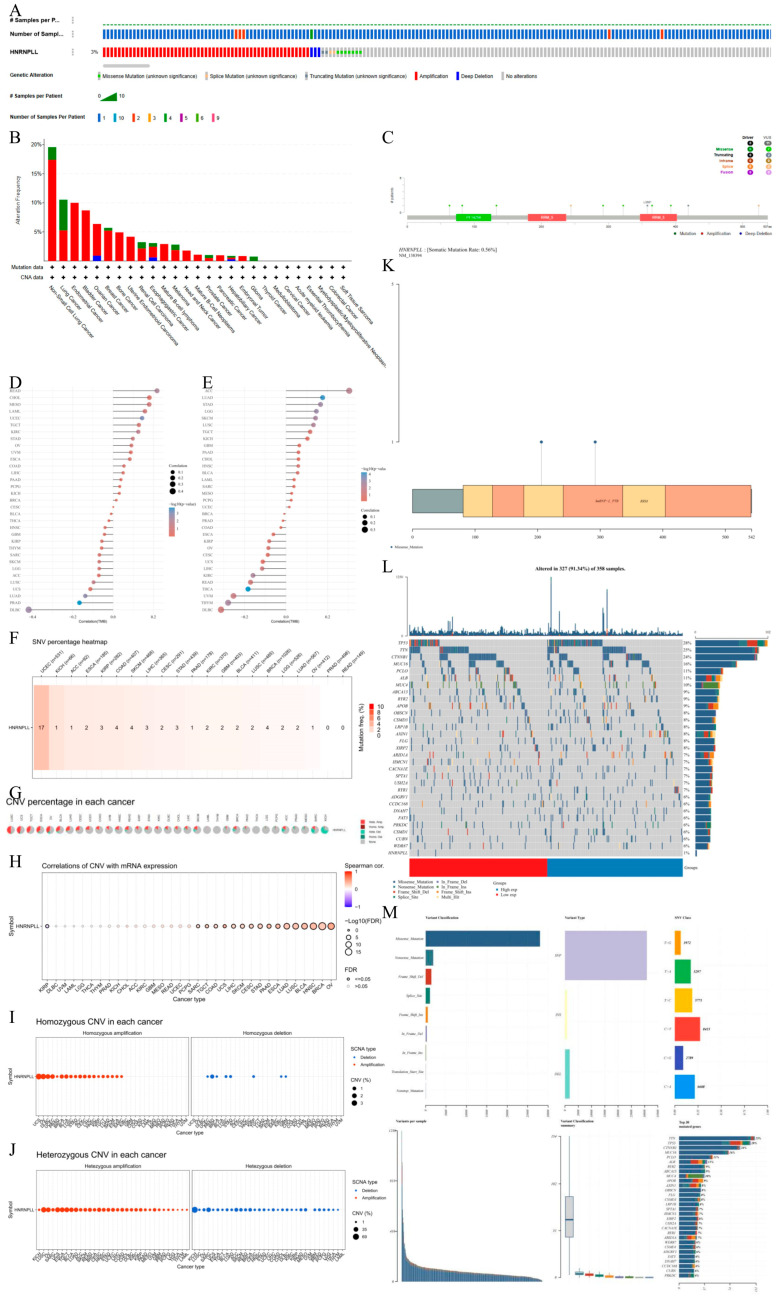
HNRNPLL mutation analysis. (**A**) Mutation status of HNRNPLL across pan-cancer types. (**B**) Alteration frequency and mutation types of HNRNPLL across pan-cancer types. (**C**) Visualization of HNRNPLL mutation sites across pan-cancer types. (**D**–**E**) Correlation analysis of HNRNPLL with TMB and MSI across pan-cancer types. (**F**) Heatmap of HNRNPLL SNV percentage in GBM and LIHC. (**G**) Pie chart of HNRNPLL CNV percentage across pan-cancer. (**H**) Bubble heatmap of HNRNPLL mRNA expression and CNV correlation across pan-cancer. (**I**) Distribution of HNRNPLL homozygous CNVs across pan-cancer. (**J**) Homozygous CNV distribution of HNRNPLL across pan-cancer. (**K**) Lollipop plot of HNRNPLL mutation distribution in LIHC. (**L**) Oncoplot of somatic mutation landscape in 358 LIHC samples. (**M**) Summary plot of variant distribution clusters by HNRNPLL variant classification, type, and SNV category.

To estimate HNRNPLL alterations across pan-cancer, we conducted a comprehensive analysis using the cBioPortal database and found that HNRNPLL was mutated in 3% of pan-cancer cases (Figure A2A). Notably, non-small-cell lung cancer, lung cancer, and endometrial cancer exhibited the highest alteration frequencies, ranking among the top three. Amplification was identified as the most prevalent type of HNRNPLL alteration; however, in lung cancer, nearly half of the alterations were missense mutations (Figure A2B). Analysis of HNRNPLL alteration positions across pan-cancer revealed 11 specific sites spanning amino acids 0 to 537 (Figure A2C). Additionally, HNRNPLL expression was positively correlated with both microsatellite instability (MSI) and tumor mutational burden (TMB) across 18 cancer types, suggesting significant implications for both TMB and MSI (Figure A2D,E). Further investigation of HNRNPLL single-nucleotide variants (SNVs) across pan-cancer yielded a heatmap of SNV frequencies, demonstrating the highest SNV ratios in uterine corpus endometrioid carcinoma (UCEC), KICH, and adrenal cortical carcinoma (ACC) (Figure A2F).

We analyzed CNVs of HNRNPLL and found that CNVs in HNRNPLL predominantly involved heterozygous amplifications across pan-cancer types (Figure A2G). HNRNPLL mRNA levels showed strong positive correlations across various tumor types, including BRCA, HNSC, OV, BLCA, and LUSC (Figure A2H). We characterized the pan-cancer CNV landscape of HNRNPLL from the perspectives of both homozygous (Figure A2I) and heterozygous (Figure A2J) amplifications and deletions. Subsequently, we performed a focused analysis of HNRNPLL alterations in LIHC and identified two alteration sites, both missense mutations, with a somatic alteration frequency of 0.56% (Figure A2K). Oncoplot analysis revealed that among 358 samples, highly altered genes such as TP53, TTN, and CTNNB1 predominantly exhibited missense mutations, with higher alteration counts in the HNRNPLL-high group (Figure A2L). We cataloged the 30 genes with the highest alteration frequencies, classified 327 mutations by alteration type and SNV category, and depicted the mutation burden for each sample based on variant classification (Figure A2M).

### 3.4. Diagnostic Importance of HNRNPLL in Pan-Cancer

**Figure 2:**
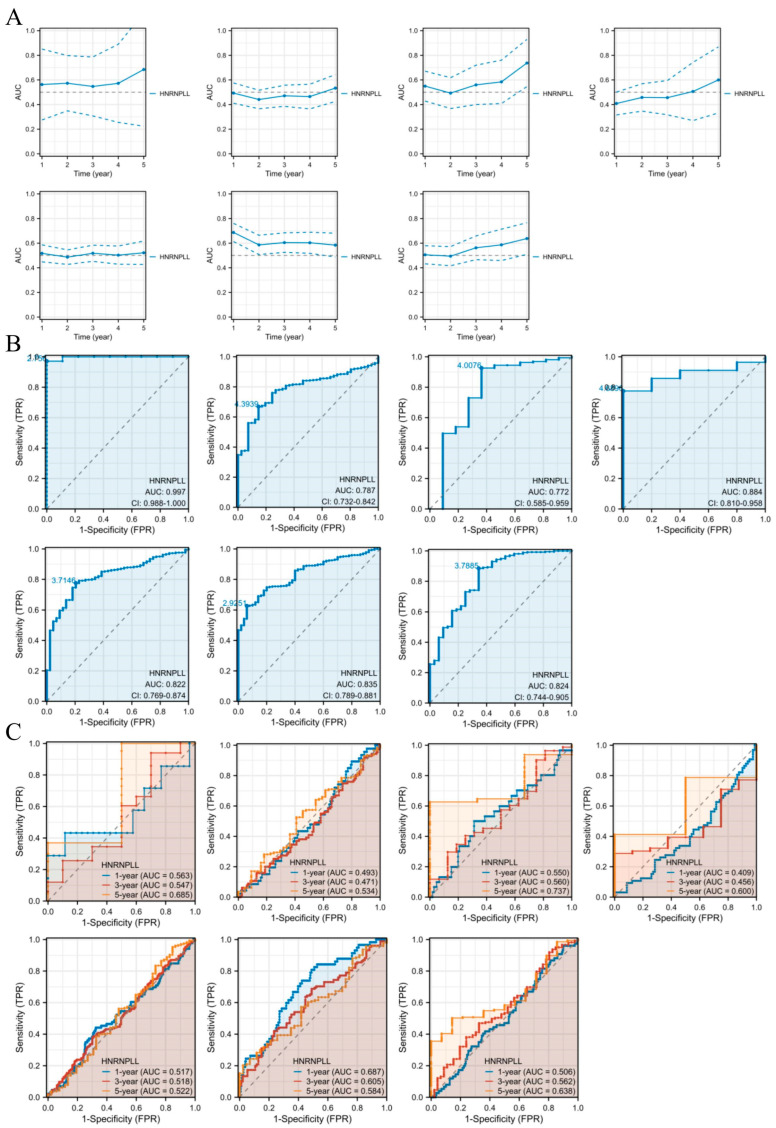
Diagnostic value of HNRNPLL across pan-cancer. (**A**) Time-dependent AUC curve for HNRNPLL. (**B**) ROC curve for HNRNPLL. (**C**) Time-dependent ROC curve for HNRNPLL.

Time-dependent AUC curves indicated that elevated HNRNPLL levels consistently predicted poorer 1- to 5-year OS in CHOL, COAD, ESCA, GBM, HNSC, LIHC, and STAD (Figure 2A). HNRNPLL demonstrated robust diagnostic value across various cancer types, including CHOL (AUC = 0.997, 95% CI: 0.988–1.000), COAD (AUC = 0.787, 95% CI: 0.732–0.842), ESCA (AUC = 0.772, 95% CI: 0.585–0.959), GBM (AUC = 0.884, 95% CI: 0.810–0.958), HNSC (AUC = 0.822, 95% CI: 0.769–0.874), LIHC (AUC = 0.835, 95% CI: 0.789–0.881), and STAD (AUC = 0.824, 95% CI: 0.744–0.905) (Figure 2B). Furthermore, time-dependent ROC analysis revealed that HNRNPLL exhibited strong predictive value for 1-year, 3-year, and 5-year OS in CHOL, COAD, ESCA, GBM, HNSC, LIHC, and STAD (Figure 2C).

### 3.5. Prognostic Significance of HNRNPLL in Pan-Cancer

**Figure 3:**
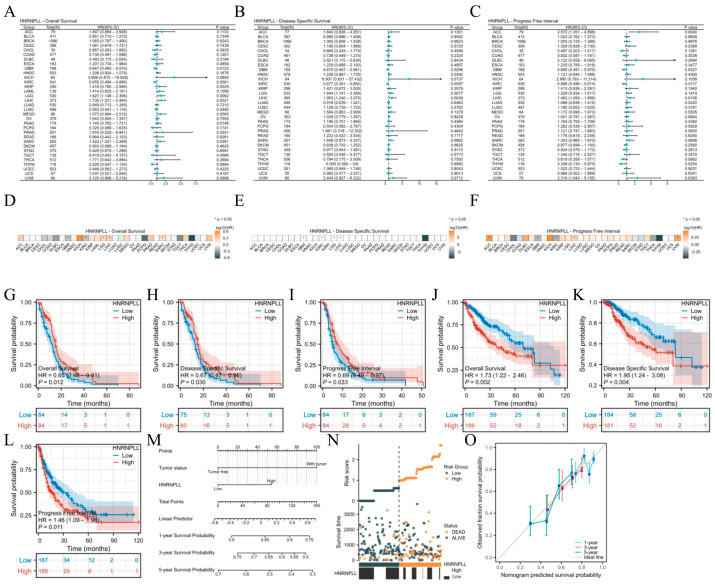
Prognostic value of HNRNPLL in pan-cancer. (**A**–**C**) Forest plots showing univariate Cox regression analysis results for HNRNPLL on OS, DSS, and PFI in TCGA pan-cancer. (**D**–**F**) Prognostic heatmaps displaying univariate Cox regression analysis results for HNRNPLL on OS, DSS, and PFI in TCGA pan-cancer. (**G**–**I**) Correlation between HNRNPLL and OS, DSS, and PFI in GBM. (**J**–**L**) Correlation between HNRNPLL and OS, DSS, and PFI in LIHC. (**M**) Prognostic nomogram incorporating HNRNPLL in LIHC. (**N**) Distribution of survival status according to HNRNPLL expression levels. (**O**) The 1-year, 3-year, and 5-year prognostic calibration curves corresponding to the HNRNPLL prognostic nomogram (0: death; 1: survival) (* *p* < 0.05).

**Figure A3:**
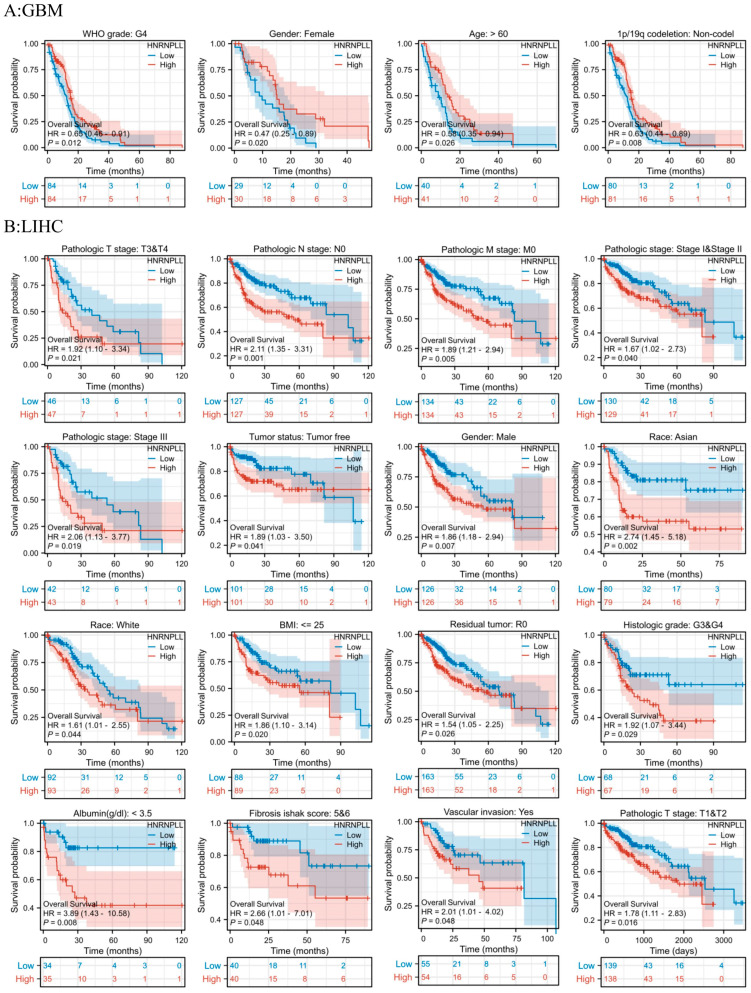
Prognostic value of HNRNPLL in GBM and LIHC subgroups. (**A**) Prognostic significance of HNRNPLL in GBM subgroups. (**B**) Prognostic significance of HNRNPLL in LIHC subgroups.

To investigate the prognostic relevance of HNRNPLL, we performed multivariate Cox regression analyses to assess its value for OS, DSS, and PFI across various cancer types. Forest plots (Figure 3A–C) and prognostic heatmaps (Figure 3D–F) illustrated the prognostic impact of HNRNPLL across different tumor subtypes. In brief, elevated HNRNPLL expression independently predicted poorer outcomes in LIHC, whereas it was associated with better prognosis in GBM and KIRC. Additionally, to validate the prognostic significance of HNRNPLL, we conducted survival analyses. High HNRNPLL expression correlated with worse outcomes in LIHC but with improved prognosis in GBM (Figure 3G–L). We further evaluated the prognostic association of HNRNPLL across various subgroups of GBM and LIHC, revealing predominantly significant prognostic correlations across all subgroups. Examples include WHO grade G4 (hazard ratio [HR] = 0.65, 95% confidence interval [CI]: 0.46–0.91, *p* = 0.012), female sex (HR = 0.47, 95% CI: 0.25–0.89, *p* = 0.020), and age > 60 years (HR = 0.58, 95% CI: 0.35–0.94, *p* = 0.026) in GBM (Figure A3A), as well as pathologic T stage T3–T4 (HR = 1.92, 95% CI: 1.10–3.34, *p* = 0.021), pathologic N stage N0 (HR = 2.11, 95% CI: 1.35–3.31, *p* = 0.001), and pathologic M stage M0 (HR = 1.89, 95% CI: 1.21–2.94, *p* = 0.005) in LIHC (Figure A3B).

In the Cox regression model, univariate Cox regression analysis revealed that pathologic T3 stage (*p* < 0.001), pathologic T4 stage (*p* < 0.001), pathologic M1 stage (*p* = 0.017), pathologic stage III–IV (*p* < 0.001), presence of tumor (*p* < 0.001), and high HNRNPLL expression (*p* = 0.002) were risk factors for OS in LIHC. Multivariate Cox regression analysis indicated that presence of tumor (*p* = 0.010) and high HNRNPLL expression (*p* = 0.035) were independent risk factors for OS in LIHC (Table 1). Based on the Cox regression results, we constructed a prognostic nomogram for LIHC incorporating HNRNPLL expression levels (Figure 3M). We developed a risk factor plot to illustrate the relationship between HNRNPLL expression levels and survival outcomes in patients with LIHC. The density of red dots representing deceased patients progressively increased with rising risk scores, indicating that elevated HNRNPLL expression was strongly correlated with increased mortality risk in LIHC (Figure 3N). The calibration curve demonstrated good concordance between predicted and actual probabilities for 1-, 3-, and 5-year OS (Figure 3O).

### 3.6. Association Between HNRNPLL Expression Levels and Clinicopathological Features in GBM and LIHC

**Figure 4:**
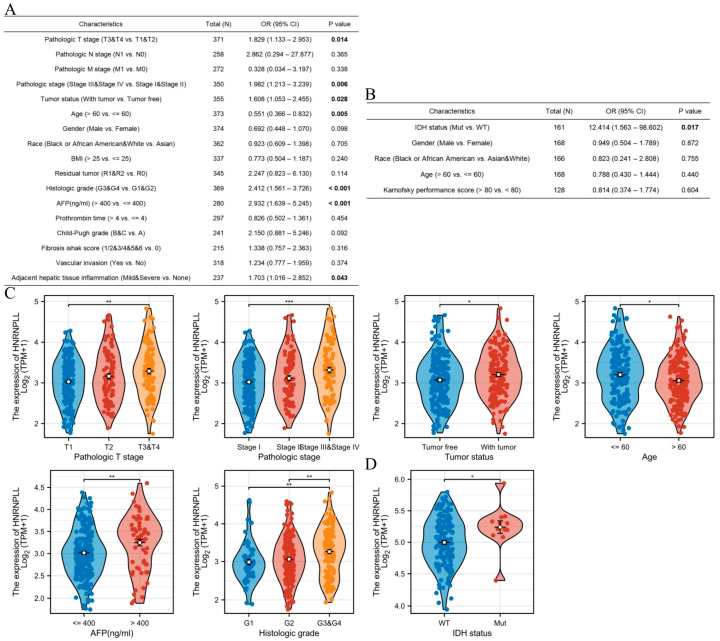
Clinical relevance of HNRNPLL in LIHC and GBM. Multivariate logistic regression analysis of HNRNPLL expression versus other clinical indicators in LIHC (**A**) and GBM (**B**). (**C**) Relationship between HNRNPLL expression and pathologic T stage, pathologic stage, tumor status, age, AFP, and histologic grade in LIHC. (**D**) Relationship between HNRNPLL expression and IDH status in GBM. (* *p* < 0.05; ** *p* < 0.01; *** *p* < 0.001).

Using multivariate logistic regression analysis, we observed that elevated HNRNPLL expression was associated with advanced pathologic T stage (odds ratio [OR] = 1.829, 95% CI: 1.113–2.953, *p* = 0.014), advanced pathologic stage (OR = 1.982, 95% CI: 1.213–3.239, *p* = 0.006), presence of tumor (OR = 1.608, 95% CI: 1.053–2.455, *p* = 0.028), age ≤ 60 years (OR = 0.551, 95% CI: 0.366–0.832, *p* = 0.005), advanced histologic grade (OR = 2.412, 95% CI: 1.561–3.726, *p* < 0.001), AFP > 400 ng/mL (OR = 2.932, 95% CI: 1.639–5.245, *p* < 0.001), and mild or severe adjacent hepatic tissue inflammation (OR = 1.703, 95% CI: 1.016–2.852, *p* = 0.043) in LIHC (Figure 4A). Similarly, elevated HNRNPLL expression correlated with mutant IDH status (OR = 12.414, 95% CI: 1.563–98.602, *p* = 0.017) in GBM (Figure 4B). In subgroup analyses, we found that elevated HNRNPLL expression was associated with advanced pathologic T stage, advanced pathologic stage, presence of tumor, age ≤ 60 years, AFP > 400 ng/mL, and advanced histologic grade in LIHC (Figure 4C). Consistently, it was associated with mutant IDH status in GBM (Figure 4D).

### 3.7. Identification of DEGs and Enrichment Analysis in GBM and LIHC

**Figure 5:**
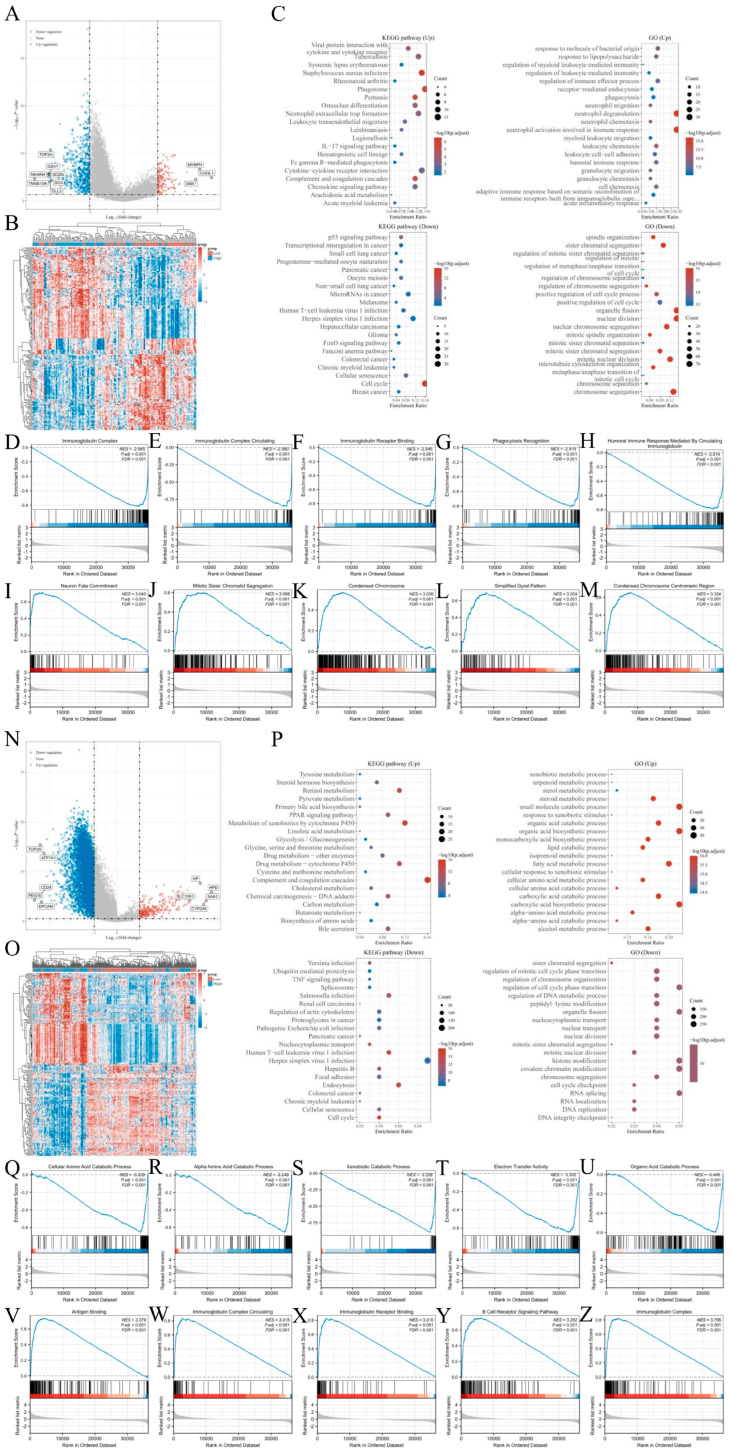
Differentially expressed gene (DEG) analysis and enrichment analysis of HNRNPLL in GBM and LIHC. (**A**) Volcano plot of DEGs in GBM. (**B**) Heatmap of DEGs in GBM. (**C**) GO and KEGG enrichment analysis of HNRNPLL-related DEGs in GBM. (**D**–**M**) GSEA enrichment analysis of down and upregulated pathways in HNRNPLL DEGs in GBM. (**N**) Volcano plot of DEGs in LIHC. (**O**) Heatmap of DEGs in LIHC. (**P**) GO and KEGG enrichment analysis of HNRNPLL DEGs in LIHC. (**Q**–**Z**) GSEA enrichment analysis of down and upregulated pathways for HNRNPLL DEGs in LIHC.

Using single-gene differential expression analysis with HNRNPLL-related genes, we identified 687 DEGs in GBM (555 downregulated and 132 upregulated) (Figure 5A,B). In LIHC, 5936 DEGs were identified (5702 downregulated and 234 upregulated) (Figure 5N,O). To characterize the functions of these genes, GO enrichment analysis and KEGG pathway analysis were performed on HNRNPLL-associated DEGs. In GBM, KEGG enrichment revealed that upregulated DEGs were significantly enriched in pathways including viral protein interaction with cytokine and cytokine receptors, tuberculosis, and systemic lupus erythematosus; downregulated DEGs were enriched in pathways such as the p53 signaling pathway, transcriptional misregulation in cancer, and small-cell lung cancer. GO enrichment showed that upregulated DEGs were significantly enriched in response to molecules of bacterial origin, response to lipopolysaccharide, and regulation of myeloid leukocyte-mediated immunity, whereas downregulated DEGs were enriched in spindle organization, sister chromatid segregation, and regulation of mitotic sister chromatid separation (Figure 5C). In LIHC, KEGG enrichment revealed that upregulated DEGs were notably enriched in pathways such as tyrosine metabolism, steroid hormone biosynthesis, and retinol metabolism; downregulated DEGs were enriched in pathways including Yersinia infection, ubiquitin-mediated proteolysis, and the TNF signaling pathway. GO enrichment indicated that upregulated DEGs were significantly enriched in xenobiotic metabolic process, terpenoid metabolic process, and sterol metabolic process, while downregulated DEGs were enriched in sister chromatid segregation, regulation of mitotic cell cycle phase transition, and regulation of chromosome organization (Figure 5P).

Next, to identify HNRNPLL-related enriched pathways, we performed GSEA. The results indicated that in GBM, the most significantly downregulated enriched pathways included the immunoglobulin complex (NES = −2.985, *p*.adj < 0.001), immunoglobulin complex circulating (NES = −2.880, *p*.adj < 0.001), immunoglobulin receptor binding (NES = −2.845, *p*.adj < 0.001), phagocytosis recognition (NES = −2.818, *p*.adj < 0.001), and humoral immune response mediated by circulating immunoglobulin (NES = −2.814, *p*.adj < 0.001). The most significantly upregulated enriched pathways included the neuron fate commitment (NES = 3.040, *p*.adj < 0.001), mitotic sister chromatid segregation (NES = 3.088, *p*.adj < 0.001), condensed chromosome (NES = 3.026, *p*.adj < 0.001), simplified gyral pattern (NES = 3.004, *p*.adj < 0.001), and condensed chromosome centromeric region (NES = 3.324, *p*.adj < 0.001) (Figure 5D–M). In LIHC, the predominantly downregulated pathways included the cellular amino acid catabolic process (NES = −3.329, *p*.adj < 0.001), alpha-amino acid catabolic process (NES = −3.249, *p*.adj < 0.001), xenobiotic catabolic process (NES = −3.229, *p*.adj < 0.001), electron transfer activity (NES = −3.302, *p*.adj < 0.001), and organic acid catabolic process (NES = −3.465, *p*.adj < 0.001); the predominantly upregulated enriched pathways included the antigen binding (NES = 3.379, *p*.adj < 0.001), immunoglobulin complex circulating (NES = 3.315, *p*.adj < 0.001), immunoglobulin receptor binding (NES = 3.310, *p*.adj < 0.001), B cell receptor signaling (NES = 3.282, *p*.adj < 0.001), and immunoglobulin complex (NES = 3.755, *p*.adj < 0.001) pathways (Figure 5Q–Z). These findings suggest that HNRNPLL may modulate downstream signal transduction by targeting these molecules, offering potential therapeutic opportunities for future clinical practice.

### 3.8. Co-Expression Genes and PPI Network of HNRNPLL in GBM and LIHC

**Figure 6:**
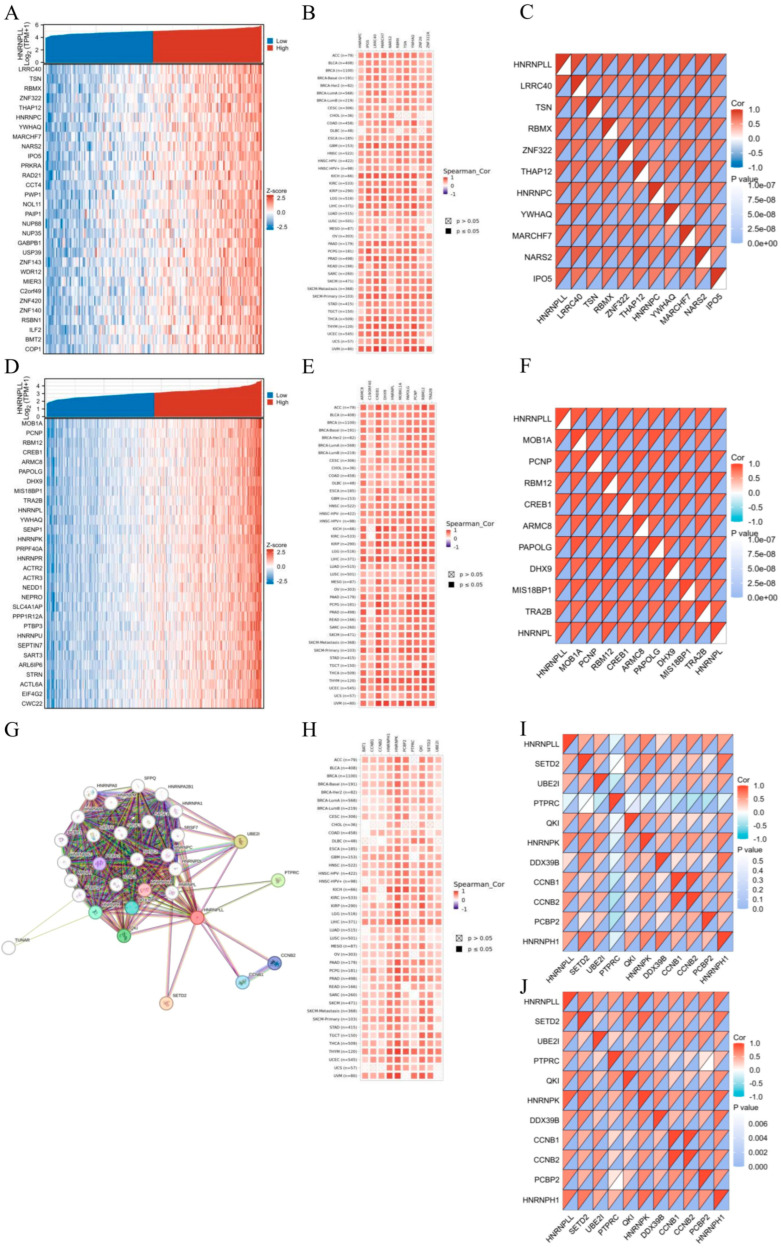
HNRNPLL-associated gene and protein interaction networks. (**A**,**D**) Heatmaps of the top 20 co-expressed genes with HNRNPLL in GBM and LIHC. (**B**,**E**) Top 10 correlation heatmaps in the GBM-LIHC co-expression network. (**C**,**F**) Correlation analysis of the top 10 genes in GBM and LIHC. (**G**) Top 20 HNRNPLL-associated proteins identified through protein interaction network analysis. (**H**) Heatmap of the top 10 associated genes in the pan-cancer protein interaction network. (**I**,**J**) Correlation analysis of the top 10 genes in GBM and LIHC.

We identified HNRNPLL co-expressed genes in GBM and LIHC using RNA sequencing data from the TCGA database and selected the 30 most significantly correlated genes (Figure 6A,D). Subsequently, we examined the expression patterns of the top 10 most significantly correlated genes across pan-cancer, most of which showed positive correlations with HNRNPLL (Figure 6B,E) and exhibited notable correlations among themselves (Figure 6C,F). Using the STRING tool, we identified 20 proteins interacting with HNRNPLL (Figure 6G). Furthermore, we evaluated the top 10 co-expressed genes with the highest interaction scores across pan-cancer and their pan-cancer correlations (Figure 6H). The results generally indicated that these genes were co-expressed in most tumors and displayed strong interrelationships among themselves in GBM (Figure 6I) and LIHC (Figure 6J).

### 3.9. Prognostic Significance of HNRNPLL Co-Expressed Genes in LIHC

**Figure 7:**
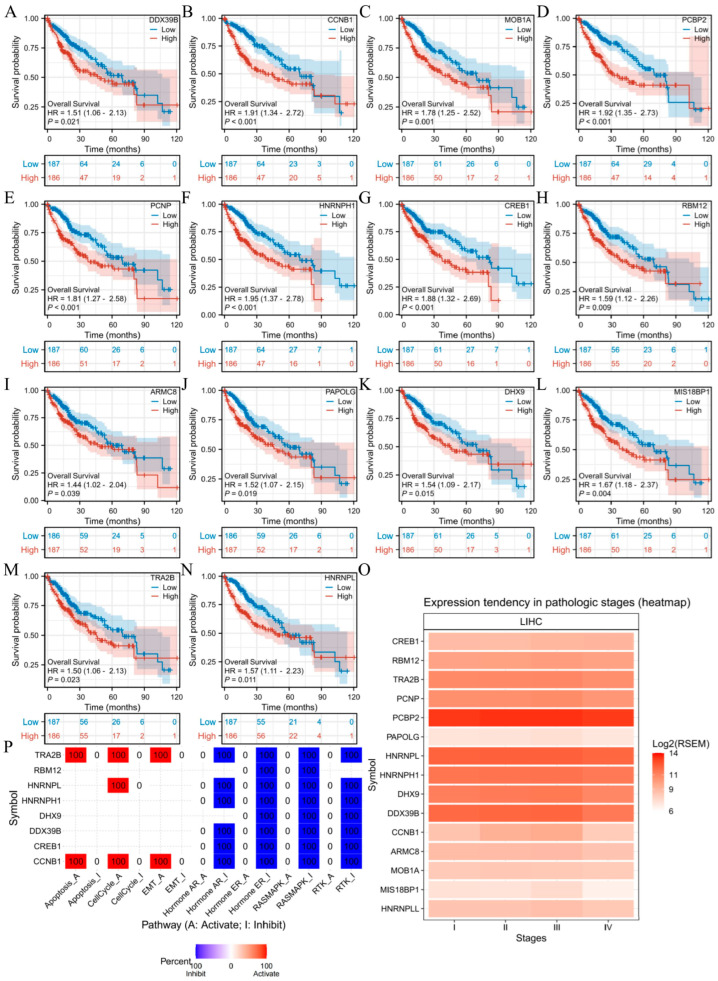
Prognostic value analysis of HNRNPLL and its co-expressed genes. (**A**–**N**) Prognostic significance of ARMC8, CCNB1, CREB1, DDX39B, DHX9, HNRNPH1, HNRNPL, MIS18BP1, MOB1A, PAPOLG, PCNP, RBM12, and TRA2B in LIHC. (**O**) Expression patterns of HNRNPLL-associated genes across different pathological stages in LIHC. (**P**) Potential common functional pathways of HNRNPLL-associated genes in LIHC.

We evaluated the prognostic significance of the aforementioned co-expressed genes and identified 14 genes with prognostic relevance in LIHC. These included DDX39B (HR = 1.51, 95% CI: 1.06–2.13, *p* = 0.021), CCNB1 (HR = 1.91, 95% CI: 1.34–2.72, *p* < 0.001), MOB1A (HR = 1.78, 95% CI: 1.25–2.52, *p* = 0.001), PCBP2 (HR = 1.92, 95% CI: 1.35–2.73, *p* < 0.001), PCNP (HR = 1.81, 95% CI: 1.27–2.58, *p* < 0.001), HNRNPH1 (HR = 1.95, 95% CI: 1.37–2.78, *p* < 0.001), CREB1 (HR = 1.88, 95% CI: 1.32–2.69, *p* < 0.001), RBM12 (HR = 1.59, 95% CI: 1.12–2.26, *p* = 0.009), ARMC8 (HR = 1.44, 95% CI: 1.02–2.04, *p* = 0.039), PAPOLG (HR = 1.52, 95% CI: 1.07–2.15, *p* = 0.019), DHX9 (HR = 1.54, 95% CI: 1.09–2.17, *p* = 0.015), MIS18BP1 (HR = 1.67, 95% CI: 1.18–2.37, *p* = 0.004), TRA2B (HR = 1.50, 95% CI: 1.06–2.13, *p* = 0.023), and HNRNPL (HR = 1.57, 95% CI: 1.11–2.23, *p* = 0.011) (Figure 7A–N). We also examined expression levels across different stages of GBM and LIHC, revealing progressively increasing expression levels of all 14 genes with LIHC advancement (Figure 7O). Using GSCALite, we explored the potential functions of HNRNPLL and these 14 genes in LIHC, suggesting that these genes may contribute to LIHC progression by modulating apoptosis, cell cycle, epithelial–mesenchymal transition (EMT), and signal transduction (Figure 7P).

### 3.10. Correlation Between HNRNPLL Expression and TIME in Pan-Cancer

**Figure 8:**
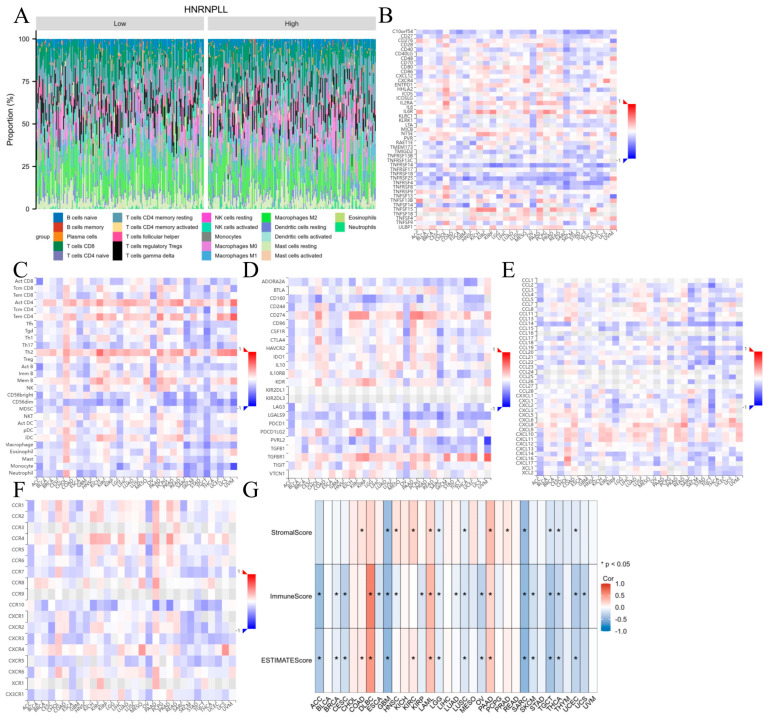
Analysis of immune cell infiltration in pan-cancer based on HNRNPLL expression. (**A**) Rainbow plot showing the distribution ratio of different immune cell types between LIHC groups with high and low HNRNPLL expression. (**B**–**F**) demonstrate the association between HNRNPLL expression and (**B**) immune stimulatory molecules, (**C**) immune cells, (**D**) immune inhibitory molecules, (**E**) chemokines, and (**F**) chemokine receptors in the TISIDB database. Red and blue represent positive and negative correlations, respectively. (**G**) Heatmap showing the correlation between HNRNPLL expression and stromal score, immune score, and ESTIMATE score across pan-cancer (* *p* < 0.05).

We initially assessed immune cell distribution across all samples using the CIBERSORT algorithm. The results revealed that in LIHC, regardless of high- or low-risk groups, immune cells in the samples were predominantly T cells and macrophages (Figure 8A). We performed gene co-expression analysis using the TISIDB database to investigate the association between HNRNPLL expression levels and various components of the tumor immune microenvironment (TIME), including immune cells, immune stimulators, immune inhibitors, chemokines, and chemokine receptors. Our analysis revealed significant correlations between HNRNPLL expression and diverse immune components across pan-cancer. Specifically, HNRNPLL expression was negatively correlated with TNFRSF25 and TNFSF14 expression in GBM (Figure 8B), with monocytes and CD56dim NK cells in uveal melanoma (UVM) (Figure 8C), with PVRL2 and TGFB1 in UVM (Figure 8D), and with CCL2, CCL22, and CXCL2 in TGCT (Figure 8E), and was negatively associated with CCR10 in pancreatic adenocarcinoma (PAAD) and prostate adenocarcinoma (PRAD) (Figure 8F). Comparative analysis of tumor stromal score, immune score, and ESTIMATE score across pan-cancer revealed strong correlations between HNRNPLL and these scores in the majority of malignancies. Elevated HNRNPLL expression was correlated with decreased tumor stromal score, immune score, and ESTIMATE score in GBM (*p* < 0.05) (Figure 8G). Collectively, these observations indicate that HNRNPLL holds broad potential for predicting immune-related phenotypes across pan-cancer.

### 3.11. Single-Cell Expression of HNRNPLL in LIHC

**Figure 9:**
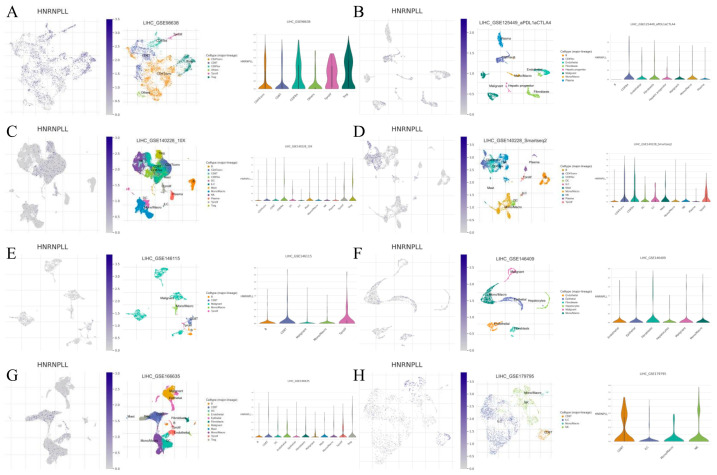
Single-cell HNRNPLL expression analysis in LIHC across GSE98638 (**A**), GSE125449_aPDL1aCTLA4 (**B**), GSE140228_10X (**C**), GSE140228_Smartseq2 (**D**), GSE146115 (**E**), GSE146409 (**F**), GSE166635 (**G**), and GSE179795 (**H**) datasets.

We evaluated the association between immune cell composition and HNRNPLL expression levels using data from eight distinct LIHC datasets in the TISCH2 database. Clustering plots revealed that HNRNPLL expression patterns varied across immune cell types, suggesting its involvement in the LIHC immune landscape. HNRNPLL expression was highest in B-cell clusters across the GSE125449_aPDL1aCTLA4, GSE140228_10X, GSE140228_Smartseq2, GSE146115, and GSE16663 datasets. HNRNPLL expression peaked in CD4+ Tconv cells (GSE98638), endothelial cells (GSE146409), and CD8+ T cells (GSE179795), respectively (Figure 9A–H).

### 3.12. Investigation of Immunotherapy and Chemotherapy Responses in Relation to HNRNPLL

**Figure 10:**
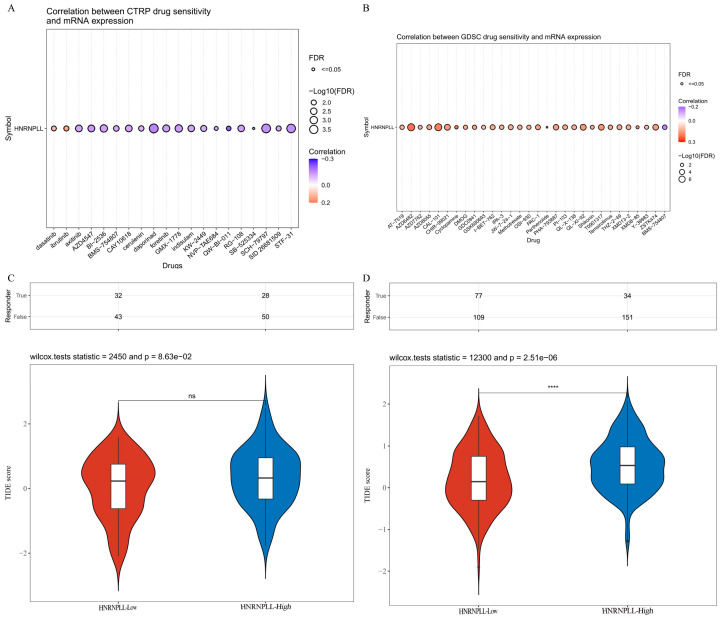
Chemotherapy drug sensitivity analysis based on HNRNPLL expression. (**A**) Correlation between CTRP drug sensitivity and HNRNPLL mRNA expression. (**B**) Correlation between GDSC drug sensitivity and HNRNPLL mRNA expression. (**C**) Violin plot of TIDE scores between high- and low-expression groups of HNRNPLL in GBM. (**D**) Violin plot of TIDE scores between high- and low-expression groups of HNRNPLL in LIHC. (ns: no significance; **** *p* < 0.0001).

Analysis of the CTRP and GDSC databases revealed that HNRNPLL exhibited negative correlations with drug sensitivity to BI-2536, daporinad, GMX-1778, SCH-79797, and STF-31, whereas it showed positive associations with AZD6482, CAL-101, CHIR-99021, QL-X1-92, and T0901317 (Figure 10A,B). To evaluate the clinical relevance of HNRNPLL in immunotherapy, we compared ICI responses between high- and low-expression samples. Although no significant difference was observed in GBM (Figure 10C), the LIHC cohort with lower HNRNPLL expression exhibited significantly reduced TIDE scores (*p* < 0.001) (Figure 10D).

### 3.13. Correlation Between HNRNPLL and Genes Associated with Ferroptosis and m6A Methylation in GBM and LIHC and Molecular Docking Analysis of HNRNPLL

**Figure 11:**
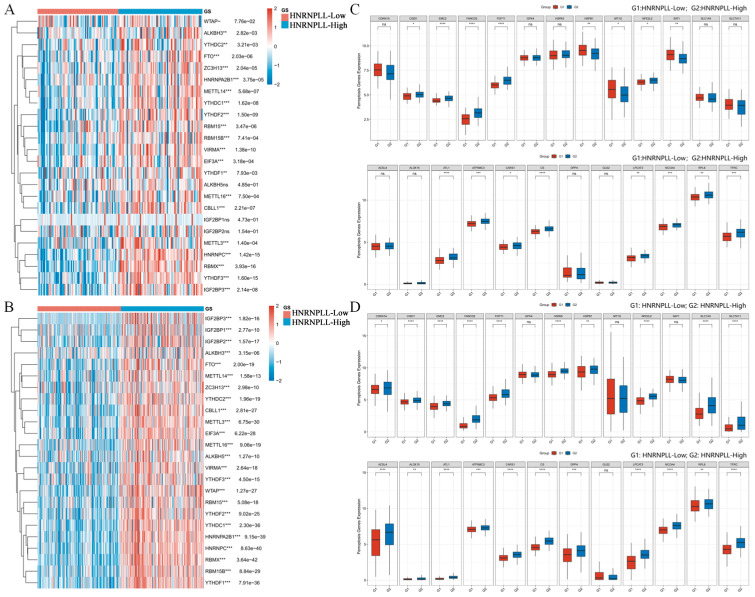
Correlation between HNRNPLL and ferroptosis-related and m6A methylation-related genes in GBM and LIHC. (**A**,**B**) Correlation between HNRNPLL and m6A methylation-related genes in GBM and LIHC. (**C**,**D**) Correlation between HNRNPLL and ferroptosis-related genes in GBM and LIHC (ns: no significance; * *p* < 0.05; ** *p* < 0.01; *** *p* < 0.001, **** *p* < 0.0001).

**Figure A4:**
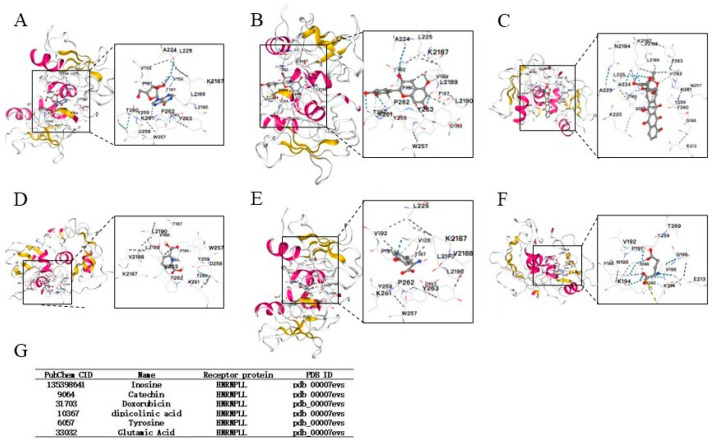
Molecular docking of HNRNPLL. Molecular docking of inosine (**A**), catechin (**B**), doxorubicin (**C**), dipicolinic acid (**D**), tyrosine (**E**), and glutamic acid (**F**) with HNRNPLL. (**G**) Overview of molecular docking with HNRNPLL.

We investigated the correlation between HNRNPLL and genes associated with ferroptosis and m6A methylation in GBM and LIHC. Regarding m6A methylation, HNRNPLL expression correlated with all m6A methylation-related genes in both malignancies, with the exception of WTAP, ALKBH5, IGF2BP1, and IGF2BP2. These included METTL3, METTL14, VIRMA, RBM15, RBM15B, ZC3H13, METTL16, CBLL1, YTHDC1, YTHDC2, YTHDF3, YTHDF1, YTHDF2, HNRNPC, IGF2BP3, RBMX, EIF3A, HNRNPA2B1, FTO, and ALKBH3 (Figure 11A,B). Regarding ferroptosis, HNRNPLL showed correlations with the majority of ferroptosis-related genes in both tumor types. Specifically, CDKN1A, HSPB1, RPL8, HSPA5, and FANCD2 in LIHC and ATL1, RPL8, CARS1, SAT1, and CISD1 in GBM were significantly correlated with HNRNPLL (Figure 11C,D). We refined the molecular docking model of HNRNPLL and identified strong docking sites with inosine, catechin, doxorubicin, dipicolinic acid, tyrosine, and glutamic acid (Figure A4A–G).

### 3.14. Validating the Biological Functions of HNRNPLL in LIHC In Vitro and In Vivo

**Figure 12:**
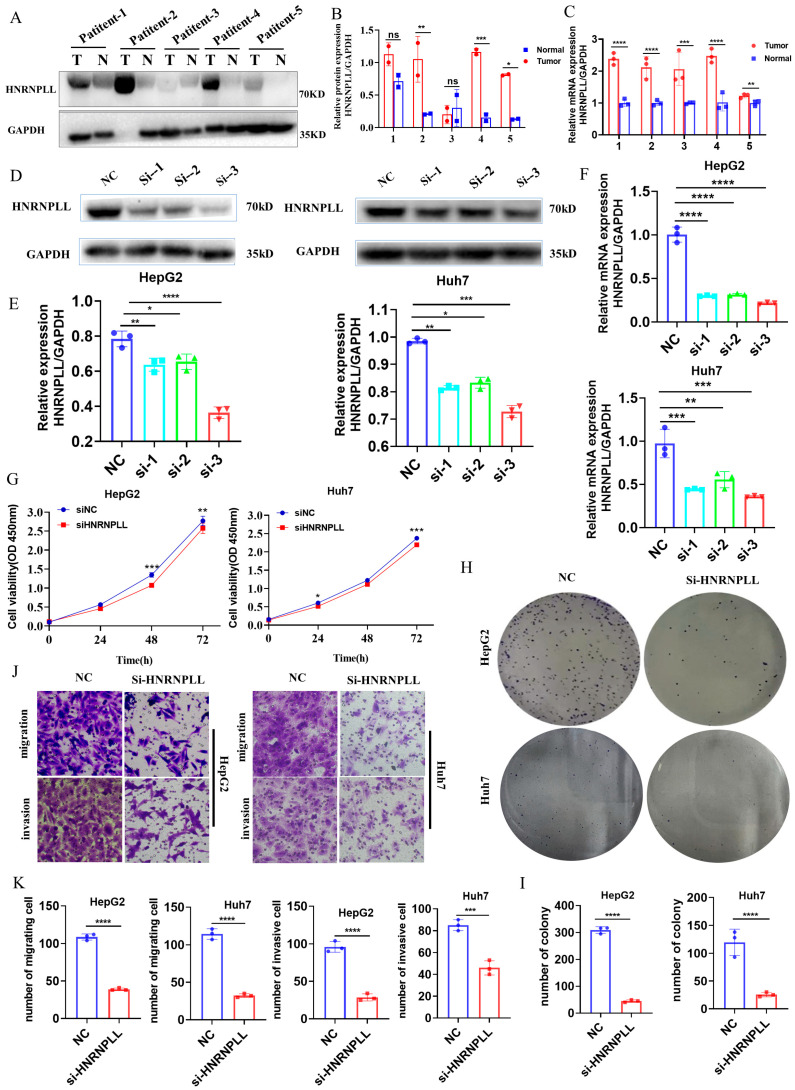
The biological function of HNRNPLL in LIHC. (**A**,**B**) Western blot was used to detect the expression levels of HNRNPLL protein in 5 pairs of LIHC clinical specimens and conduct quantification. (T: tumor, N: normal.) Full-length blots are included in the Appendix A. (**C**) RT-qPCR detection of HNRNPLL mRNA expression levels in specimens from 5 pairs of LIHC patients. (**D**,**E**) Western blot assay was performed after knockdown of HNRNPLL in HepG2 and Huh-7 cells. Full-length blots are included in the Appendix A. (**F**) RT-qPCR was used to detect the expression levels of HNRNPLL mRNA in HepG2 and Huh-7 cells after knockdown of HNRNPLL. (**G**) CCK-8 assay to investigate the relationship between HNRNPLL expression and growth ability. (**H**,**I**) Colony formation assay was performed to determine the relationship between HNRNPLL expression and clone formation ability. (**J**,**K**) Migration and invasion assays were conducted to determine the relationship between HNRNPLL expression and cell migration and invasion ability. (ns: no significance; * *p* < 0.05; ** *p* < 0.01; *** *p* < 0.001, **** *p* < 0.0001).

The biological importance of HNRNPLL prompted us to conduct experimental validation. Initially, Western blot analysis of LIHC tissues and matched normal controls suggested higher expression of HNRNPLL in LIHC (Figure 12A,B). This finding was corroborated by qRT-PCR, which also indicated elevated HNRNPLL levels in LIHC (Figure 12C). To investigate its functional role, we performed siRNA-mediated knockdown of HNRNPLL in HepG2 and Huh7 cells. Knockdown efficiency was verified by Western blot and qPCR, with the third siRNA construct (si-HNRNPLL-3) proving most effective and thus chosen for further study (Figure 12D–F). Functional assays revealed that HNRNPLL depletion suppressed cell proliferation, as measured by CCK-8 (Figure 12G), and reduced colony-forming capacity (Figure 12H,I). Additionally, both migration and invasion were significantly attenuated in HNRNPLL-deficient cells (Figure 12J,K). In conclusion, these data establish a key role for HNRNPLL in modulating proliferation, migration, and invasion of HepG2 and Huh7 cells in vitro.

**Figure 13:**
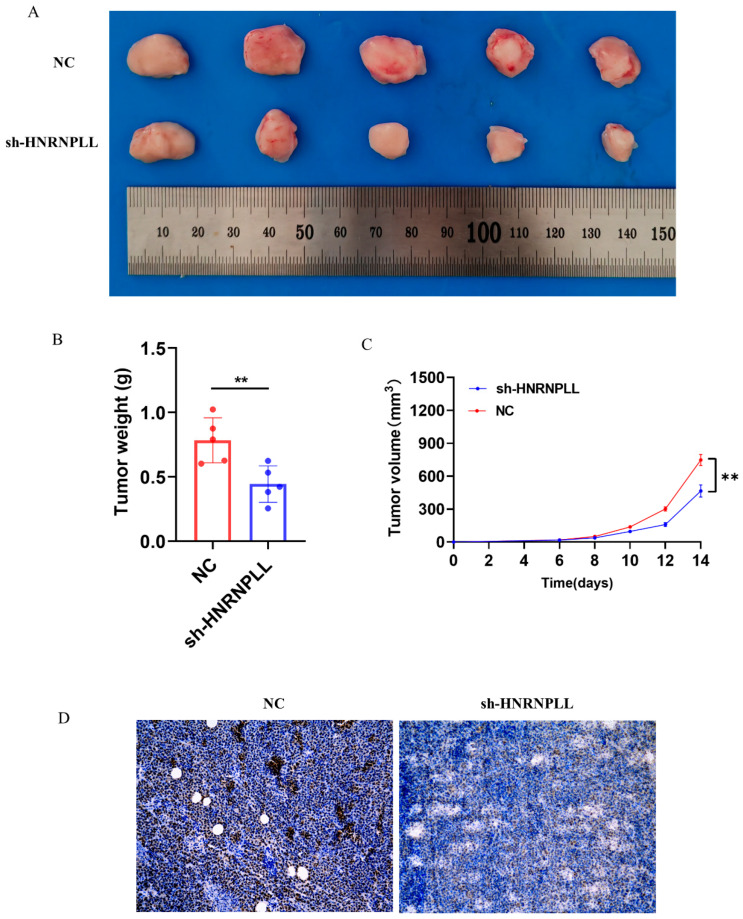
HNRNPLL promotes the proliferation of HepG2 cells in vivo. (**A**) In subcutaneous tumorigenesis experiments with nude mice, the size of the tumor was detected after HNRNPLL knockdown. (**B**) The weight of the tumor was detected after HNRNPLL knockdown. (**C**) The volume of the tumor was detected after HNRNPLL knockdown after 14 days. (**D**) Ki67 immunohistochemical analysis of the proliferative activity of tumor tissues after HNRNPLL knockdown. (** *p* < 0.01).

To investigate the oncogenic role of HNRNPLL in vivo, we performed a subcutaneous xenograft assay using HepG2 cells in 6-week-old nude mice. Tumor growth was monitored by volume measurements every other day until termination at day 14. The results indicated a marked reduction in tumor size, volume, and final weight upon HNRNPLL knockout (Figure 13A–C). Consistent with this phenotype, Ki67 immunohistochemical staining showed a concomitant decrease in proliferating cells within the HNRNPLL-depleted tumors (Figure 13D).

## 4. Discussion

In the present study, we combined integrative multi-omics analyses with experimental validation to investigate the potential role of HNRNPLL in LIHC. Our results indicate that HNRNPLL is significantly upregulated in LIHC and associated with unfavorable clinicopathological features and poor survival outcomes. Functional assays further suggest that HNRNPLL may contribute to tumor cell proliferation, migration, and invasion, supporting its potential involvement in LIHC progression.

RNA-binding proteins have emerged as critical regulators of post-transcriptional gene expression, and their dysregulation has been increasingly implicated in tumorigenesis [44]. Members of the hnRNP family are known to participate in diverse cancer-related processes, including RNA splicing, cell cycle regulation, and immune modulation [10,45,46]. As a member of this family, HNRNPLL has been primarily studied in immune cell differentiation and RNA processing [47]. Our findings extend these observations by suggesting that HNRNPLL may also be associated with tumor-related biological processes in LIHC.

Functional enrichment analyses revealed that genes associated with HNRNPLL expression are enriched in pathways related to cell cycle progression, mitotic regulation, and epithelial–mesenchymal transition, which are well-established drivers of tumor growth and metastasis [48,49]. These findings are consistent with our experimental results showing that HNRNPLL knockdown suppresses proliferation and invasive behavior in LIHC cell lines. However, it should be noted that enrichment analyses are based on statistical associations and do not establish causal relationships.

In addition, our analyses identified significant correlations between HNRNPLL expression and features of the tumor immune microenvironment, including immune cell infiltration and immune-related signaling pathways. These findings suggest that HNRNPLL may be linked to immune-related processes in LIHC [50,51,52]. However, these observations are derived from computational deconvolution methods and were not experimentally validated in this study. Therefore, the role of HNRNPLL in immune regulation remains to be further clarified.

We also observed correlations between HNRNPLL and genes involved in ferroptosis and m6A methylation. These findings may indicate a broader involvement of HNRNPLL in transcriptomic and metabolic regulation. Nevertheless, these associations should be interpreted cautiously, as no direct functional experiments were performed to validate these relationships.

In addition, several limitations inherent to pan-cancer bioinformatic analyses should be considered. First, most of the datasets used in this study were derived from public databases such as TCGA and GEO, which may be affected by batch effects, differences in sample processing, and variability in data acquisition across platforms. Although normalization procedures were applied, residual heterogeneity cannot be completely eliminated. Second, pan-cancer analyses integrate data from multiple tumor types with distinct biological characteristics. Therefore, associations identified at the pan-cancer level may not fully reflect tumor-specific mechanisms, including those in LIHC. Third, many findings in this study are based on correlation analyses, which do not imply causality. The observed relationships between HNRNPLL expression and immune features, ferroptosis-related genes, and m6A methylation regulators may reflect indirect or co-regulated processes rather than direct regulatory effects. Finally, computational methods used for immune infiltration estimation and pathway analysis rely on predefined algorithms and reference datasets, which may introduce methodological bias. These limitations should be considered when interpreting the results.

Beyond these methodological considerations, several additional limitations should be acknowledged. First, the number of clinical samples used for experimental validation was relatively small, which may limit the generalizability of the findings. Second, the in vivo experiments were conducted with a limited sample size, which may reduce statistical power. Third, although extensive bioinformatic analyses were performed, the mechanistic role of HNRNPLL in LIHC progression was not directly investigated. Future studies with larger cohorts and more in-depth mechanistic experiments are required to validate and extend these findings.

Importantly, it remains possible that increased HNRNPLL expression reflects broader alterations in cellular proliferation or RNA metabolism rather than acting as a direct oncogenic driver. This alternative explanation should be considered in future studies.

In conclusion, our study suggests that HNRNPLL is associated with LIHC progression and tumor-related biological processes. While our findings highlight its potential as a prognostic biomarker and candidate therapeutic target, further studies are required to elucidate its precise biological functions and clinical relevance.

## Figures and Tables

**Table 1:** The COX regression results in LIHC.

Characteristics	Total(N)	Univariate Analysis		Multivariate Analysis	
		Hazard Ratio (95% CI)	*p* Value	Hazard Ratio (95% CI)	*p* Value
Pathologic T stage	370				
T1	183	Reference		Reference	
T2	94	1.431 (0.902–2.268)	0.128	1.646 (0.901–3.007)	0.105
T3	80	2.674 (1.761–4.060)	**<0.001**	2.043 (0.268–15.589)	0.491
T4	13	5.386 (2.690–10.784)	**<0.001**	3.528 (0.396–31.418)	0.258
Pathologic N stage	258				
N0	254	Reference			
N1	4	2.029 (0.497–8.281)	0.324		
Pathologic M stage	272				
M0	268	Reference		Reference	
M1	4	4.077 (1.281–12.973)	**0.017**	1.076 (0.198–5.853)	0.933
Pathologic stage	349				
Stage I and Stage II	259	Reference		Reference	
Stage III and Stage IV	90	2.504 (1.727–3.631)	**<0.001**	1.376 (0.187–10.138)	0.754
Residual tumor	344				
R0	326	Reference			
R1&R2	18	1.604 (0.812–3.169)	0.174		
Histologic grade	368				
G1	55	Reference			
G2	178	1.162 (0.686–1.969)	0.576		
G3	123	1.185 (0.683–2.057)	0.545		
G4	12	1.681 (0.621–4.549)	0.307		
Gender	373				
Female	121	Reference			
Male	252	0.793 (0.557–1.130)	0.200		
Age	373				
≤60	177	Reference			
>60	196	1.205 (0.850–1.708)	0.295		
Race	361				
Asian	159	Reference			
Black or African American	17	1.585 (0.675–3.725)	0.290		
White	185	1.323 (0.909–1.928)	0.144		
Tumor status	354				
Tumor free	202	Reference		Reference	
With tumor	152	2.317 (1.590–3.376)	**<0.001**	1.845 (1.154–2.950)	**0.010**
BMI	336				
≤25	177	Reference			
>25	159	0.798 (0.550–1.158)	0.235		
AFP(ng/mL)	279				
≤400	215	Reference			
>400	64	1.075 (0.658–1.759)	0.772		
Prothrombin time	296				
≤4	207	Reference			
>4	89	1.335 (0.881–2.023)	0.174		
Albumin(g/dL)	299				
<3.5	69	Reference			
≥3.5	230	0.897 (0.549–1.464)	0.662		
Child–Pugh grade	240				
A	218	Reference			
B&C	22	1.643 (0.811–3.330)	0.168		
Ishak fibrosis score	214				
0	75	Reference			
1/2&3/4&5&6	139	0.772 (0.465–1.281)	0.316		
Vascular invasion	317				
No	208	Reference			
Yes	109	1.344 (0.887–2.035)	0.163		
Adjacent hepatic tissue inflammation	236				
None	118	Reference			
Mild	101	1.204 (0.723–2.007)	0.476		
Severe	17	1.144 (0.447–2.930)	0.779		
HNRNPLL	373				
Low	187	Reference		Reference	
High	186	1.735 (1.221–2.464)	**0.002**	1.641 (1.036–2.598)	**0.035**

## Data Availability

All data generated or analyzed during this study are included in this published article.

## Abbreviations

hnRNP	heterogeneous nuclear ribonucleoprotein
LIHC	liver hepatocellular carcinoma
GBM	glioblastoma
AFP	alpha-fetoprotein
SEER	surveillance, epidemiology, and end results
OS	overall survival
HNRNPLL	heterologous nuclear ribonucleoprotein L-like protein
hnRNP L	heterologous nuclear ribonucleoprotein L
Ptprc	tyrosine phosphatase receptor C
TCGA	Cancer Genome Atlas
GEO	Gene Expression Omnibus
HPA	Human Proteome Atlas
PPI	Protein–protein interaction
DEGs	differentially expressed genes
IHC	immunohistochemistry
TMB	tumor mutational burden
MSI	microsatellite instability
CNV	copy number variation
ROC	receiver operating characteristic
AUC	area under the curve
DSS	disease-specific survival
PFI	progression-free interval
PT	prothrombin time
*p*.adj	adjusted *p*-value
GO	Gene Ontology
KEGG	Kyoto Encyclopedia of Genes and Genomes
GSEA	Gene Set Enrichment Analysis
NES	normalized enrichment score
TIMER2	Tumor Immune Assessment Resource 2.0
TIME	tumor immune microenvironment
TISCH	Tumor Immune Single-Cell Hub
t-SNE	t-distributed stochastic neighbor embedding
TIDE	tumor immune dysfunction and exclusion
ICI	immune checkpoint inhibitor
GDSC	genomic drug sensitivity in cancer
CTRP	Cancer Therapy Response Portal
IC50	half-maximal inhibitory concentration
RT-qPCR	quantitative real-time PCR
si-HNRNPLL-3	selected HNRNPLL-targeting siRNA
CCK-8	Cell Counting Kit-8
ARRIVE	animal research: reporting of in vivo experiments
ANOVA	analysis of variance
SD	standard deviation
CHOL	cholangiocarcinoma
COAD	colorectal adenocarcinoma
ESCA	esophageal carcinoma
HNSC	head and neck squamous cell carcinoma
STAD	stomach adenocarcinoma
KICH	kidney chromophobe
THCA	thyroid carcinoma
CRC	colorectal cancer
BRCA	breast cancer
SNVs	single-nucleotide variants
UCEC	uterine corpus endometrioid carcinoma
ACC	adrenal cortical carcinoma
HR	hazard ratio
CI	confidence interval
OR	odds ratio
UVM	uveal melanoma
PAAD	pancreatic adenocarcinoma
PRAD	prostate adenocarcinoma
MSCs	mesenchymal stem-like cells
PD-1	programmed death-1
PD-L1	programmed cell death-ligand 1
CTLA-4	cytotoxic T-lymphocyte-associated protein 4
